# Investigating the genetic profile of dopaminergic neurons in the VTA in response to perinatal nicotine exposure using mRNA-miRNA analyses

**DOI:** 10.1038/s41598-018-31882-9

**Published:** 2018-09-13

**Authors:** Renee F. Keller, Andrei Dragomir, Fan Yantao, Yasemin M. Akay, Metin Akay

**Affiliations:** University of Houston, Department of Biomedical Engineering, Houston, TX 77204 USA

## Abstract

Maternal smoking during pregnancy is associated with an increased risk of developmental, behavioral, and cognitive deficits. Nicotine, the primary addictive component in tobacco, has been shown to modulate changes in gene expression when exposure occurs during neurodevelopment. The ventral tegmental area (VTA) is believed to be central to the mechanism of addiction because of its involvement in the reward pathway. The purpose of this study was to build a genetic profile for dopamine (DA) neurons in the VTA and investigate the disruptions to the molecular pathways after perinatal nicotine exposure. Initially, we isolated the VTA from rat pups treated perinatally with either nicotine or saline (control) and collected DA neurons using fluorescent-activated cell sorting. Using microarray analysis, we profiled the differential expression of mRNAs and microRNAs from DA neurons in the VTA in order to explore potential points of regulation and enriched pathways following perinatal nicotine exposure. Furthermore, mechanisms of miRNA-mediated post-transcriptional regulation were investigated using predicted and validated miRNA-gene targets in order to demonstrate the role of miRNAs in the mesocorticolimbic DA pathway. This study provides insight into the genetic profile as well as biological pathways of DA neurons in the VTA of rats following perinatal nicotine exposure.

## Introduction

Maternal smoking during pregnancy is associated with adverse birth outcomes including stillbirth and low birth weight as well as sudden infant death syndrome (SIDS). In addition, many developmental abnormalities are associated with gestational nicotine exposure including behavioral disorders like attention deficit hyperactivity disorder (ADHD), learning disabilities, cognitive dysfunction, and a predisposition towards smoking later in life^1,2^. Studies have shown that exposure to nicotine, the biologically active substance in tobacco, changes the intensity and timing of brain cell development, and also the programming of neurodevelopmental events on a cellular level^3,4^. During pregnancy, nicotine readily diffuses across the placental barrier entering the fetal blood and amniotic fluid. After birth, the offspring continue to receive nicotine through breast milk, which contains two to three times more nicotine than the mother’s plasma^5^.

Nicotine activates dopaminergic (DA) neurons of the mesocorticolimbic dopamine pathway, also known as the reward pathway, which consists of the ventral tegmental area (VTA), the prefrontal cortex (PFC), the nucleus accumbens (NAc), and other limbic areas. The VTA is believed to be central to the neural adaptations that underlie addiction^6–9^. Several studies have been conducted on gestational nicotine exposure using a subcutaneously implanted osmotic minipump for nicotine treatment in order to investigate biochemical, behavioral, and genetic changes that occur due to nicotine exposure during pregnancy. In the brain, a diminished response to nicotine demonstrated by lowered levels of DA in the NAc and striatum has been found in offspring exposed to gestational nicotine or comorbid gestational nicotine and ethanol^10–12^. Additionally, several receptor subunits have been found to be modulated by gestational nicotine exposure, including nicotinic acetylcholine receptors and glutamate receptors, within the VTA DA system^11–14^.

Because of the importance of the VTA in addiction, several studies have been conducted to determine genetic profiles of the VTA in response to exposure from different addictive substances^15–17^. These studies focused on the VTA in its entirety, providing a profile of the overall area, but do not separate the contributions of different cell types to the underlying mechanism of addiction. Other studies have investigated different regions that directly or indirectly receive input from the VTA in order to identify genetic changes^18–22^. Additionally, studies have investigated gender differences present after perinatal nicotine exposure. Despite extensive research on addiction, the mechanisms remain unclear. In contrast to previous studies, we sought to focus our experiment by investigating changes that occur specifically in DA neurons in the VTA in response to perinatal nicotine exposure.

Recently, several studies have focused on the influence of microRNAs (miRNAs) in addiction^23–26^. miRNAs are short, non-coding RNA sequences that post-transcriptionally regulate genes by directly targeting the 3′-untranslated region (3′-UTR) of mRNA. In addition, several studies have also been conducted integrating the analysis of mRNA and miRNA in order to elucidate the regulatory interactions that occur following exposure to substances of abuse^27–29^.

Perinatal nicotine exposure is the most common approach to study the association between maternal smoking during pregnancy and associated disorders^4^. Therefore, given the importance of VTA DA neurons and the potential regulatory role of miRNAs, in this study we investigated the genetic profiles and biological pathways of DA neurons in the VTA in response to perinatal nicotine exposure using high-throughput microarray analyses of mRNA and miRNA expressions.

## Results

The VTA was isolated from rat pups treated perinatally with nicotine or saline (control). After dissociating and sorting our samples, an average of 27.9% ± 3.3% of the NeuN-positive neurons were positively stained by TH. These results confirm the proportion of double-stained neurons reported by Guez-Barber *et al*., who found that 30.2% of all NeuN-positive events were double stained for TH and NeuN from midbrain samples. Another study by Chung *et al*. followed the same protocol and reported approximately 25% of NeuN-positive cells were also TH-positive^30^. The exact percentage of TH-positive neurons in the VTA is not known, but is speculated to be about 50–60%^31^; therefore, we considered the estimated 25–30% of TH-positive neurons after fluorescent-activated cell sorting (FACS) to be adequate for the suggested experiment, consistent with other studies^30,32^. There was no significant difference in the number of collected DA neurons per VTA between the treatment groups since the saline-treated and nicotine-treated groups had 27.1% ± 3.7% and 28.5% ± 3.1% double-positive neurons from the total NeuN-positive neurons, respectively. Furthermore, statistical analysis using Student’s t-test showed no significant difference (p > 0.5).

After collecting DA neurons using FACS, samples were processed using mRNA and miRNA microarrays in order to find the differential expression profiles of DA neurons after perinatal exposure to nicotine. The transcriptome and miRNome were then used to identify potential points of regulation and enriched pathways following perinatal nicotine exposure in VTA DA neurons.

### mRNA expression changes following perinatal nicotine exposure

**Figure 1:**
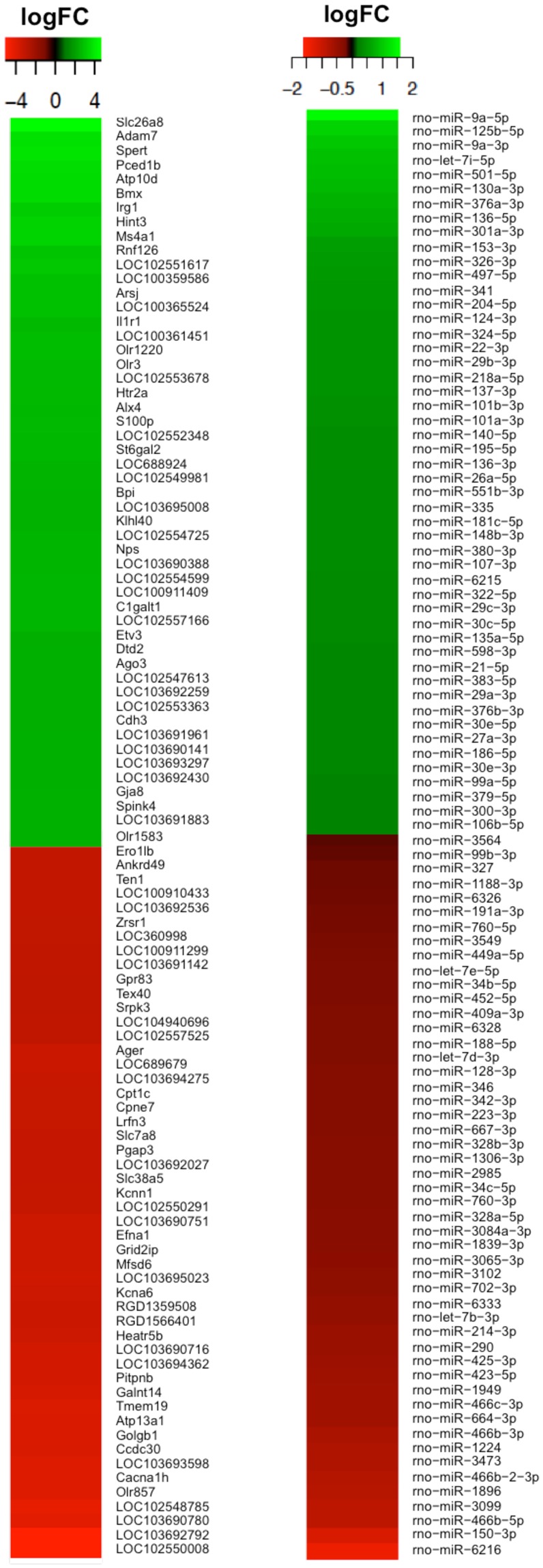
Heat maps of DEGs (left) and DEmiRs (right) in DA neurons in the VTA following perinatal nicotine exposure compared to saline control. Displayed are expression profiles of the most altered genes and miRNAs based on greatest absolute log fold change selected from the microarrays. Red denotes decreased expression; while green denotes increased expression.

For VTA DA neurons, 2,636 genes were found to be differentially expressed at a q-value < 0.05 using Benjamini & Hochberg (BH) method and an absolute log2 fold change >1. More specifically, 862 differentially expressed genes (DEGs) were upregulated and 1,774 DEGs were downregulated showing that the majority of genes in VTA DA neurons are downregulated due to nicotine exposure. Of these genes, 2,095 were annotated genes (639 upregulated and 1,456 downregulated). Figure 1 shows a heatmap of the top overall results for up and downregulated genes in DA neurons. Details about the top up and downregulated genes are shown in Table 1.

**Table 1:** Top 20 DEGs based on p-value from microarray expression analysis of DA neurons following perinatal nicotine exposure compared to saline control. Predicted miRNA targets are also included.

Gene Symbol	Entrez ID	Log FC	adj *p* val	Description	miRNA target
*Upregulated*					
Nepn	309775	1.635	0.00093	nephrocan	
Spert	498572	3.824	0.00118	spermatid associated	
Map2	25595	1.365	0.00124	microtubule-associated protein 2	
Pced1b	315283	3.652	0.00138	PC-esterase domain containing 1B	
LOC103691883	103691883	2.475	0.00153	uncharacterized LOC103691883	
Sept11	305227	1.017	0.00166	septin 11	rno-miR-30b-5p, rno-miR-30d-5p
Ptp4a2	85237	1.114	0.00175	protein tyrosine phosphatase type IVA, member 2	rno-miR-290
S100a1	295214	1.243	0.00175	S100 calcium binding protein A1	
LOC102547613	102547613	2.412	0.00175	uncharacterized LOC102547613	
LOC103691014	103691014	1.545	0.00175	uncharacterized LOC103691014	
Rnf126	314613	2.96	0.00186	ring finger protein 126	
LOC103693092	103693092	2.542	0.00191	uncharacterized LOC103693092	
Sepp1	29360	1.164	0.00194	selenoprotein P, plasma, 1	
Lrrc25	498605	1.536	0.00208	leucine rich repeat containing 25	
Nkain3	689576	1.466	0.00219	Na + /K + transporting ATPase interacting 3	
Bcas1	246755	1.46	0.00240	breast carcinoma amplified sequence 1	rno-miR-214-3p
Bcl11a	305589	2.137	0.00242	B-cell CLL/lymphoma 11 A (zinc finger protein)	
Bmx	367786	3.555	0.00242	BMX non-receptor tyrosine kinase	
Gpm6b	192179	1.26	0.00245	glycoprotein m6b	
LOC102553223	102553223	1.118	0.00245	uncharacterized LOC102553223	
*Downregulated*					
LOC103692792	103692792	−5.074	0.00014	inactive phospholipase C-like protein 2	
Pfkp	60416	−2.062	0.00041	phosphofructokinase, platelet	
Atp13a1	290673	−3.882	0.00041	ATPase type 13A1	rno-miR-335
LOC102546798	102546798	−2.342	0.00041	uncharacterized LOC102546798	
LOC102557363	102557363	−2.301	0.00041	uncharacterized LOC102557363	
Cldn11	84588	−1.783	0.00048	claudin 11	rno-miR-500-3p
Cstf2	683927	−2.386	0.00050	cleavage stimulation factor, 3′ pre-RNA subunit 2	
LOC102546798	102546798	−1.664	0.00055	uncharacterized LOC102546798	
Agpat4	170919	−1.539	0.00058	1-acylglycerol-3-phosphate O-acyltransferase 4	
LOC100359748	100359748	−2.02	0.00058	zinc finger CCCH type, antiviral 1	
Mfsd6	301388	−3.487	0.00070	major facilitator superfamily domain containing 6	rno-miR-30b/e-5p, rno-miR-301a-3p, rno-miR-130a-3p, rno-miR-26a-5p, rno-miR-101a/b-3p
LOC102554532	102554532	−2.769	0.00070	uncharacterized LOC102554532	
LOC102555987	102555987	−2.624	0.00070	uncharacterized LOC102555987	
Snrpn	81781	−2.153	0.00077	small nuclear ribonucleoprotein polypeptide N	
LOC103693598	103693598	−3.972	0.00077	uncharacterized LOC103693598	
Pgap3	688174	−3.218	0.00079	post-GPI attachment to proteins 3	
LOC102557525	102557525	−3.072	0.00079	disks large homolog 5-like	
Uchl1	29545	−1.425	0.00085	ubiquitin carboxyl-terminal esterase L1 (ubiquitin thiolesterase)	
Kcnt1	60444	−2.582	0.00093	potassium channel, sodium-activated subfamily T, member 1	rno-miR-324-5p
Slc7a8	84551	−3.219	0.00093	solute carrier family 7 (amino acid transporter light chain, L system), member 8	

### miRNA expression changes following perinatal nicotine exposure

For DA neurons, there were 74 differentially expressed miRNAs (DEmiRs) of which 58 miRNAs were found to be upregulated, while 16 were downregulated showing that the majority of miRNAs were upregulated due to perinatal nicotine exposure (see Fig. 1 for heatmap). Applying parameters *q*-value < 0.05 (BH) and an absolute log2 fold change > 0.5^29^, we found 11 DEmiRs that were upregulated and 9 DEmiRs that were downregulated. DEmiR results are shown in Table 2.

**Table 2:** Significantly differentially expressed miRNAs (DEmiRs) in DA neurons of the VTA following perinatal nicotine exposure.

miRNA Accession	miRNA name	logFC	adj *p* val	miRNA Accession	miRNA name	logFC	adj *p* val
*Upregulated*							
MIMAT0000836	rno-miR-130a-3p	0.862	0.00001	MIMAT0005341	rno-miR-488-3p	0.131	0.01457
MIMAT0000779	rno-let-7i-5p	0.912	0.00001	MIMAT0000888	rno-miR-218a-5p	0.453	0.01460
MIMAT0000830	rno-miR-125b-5p	1.106	0.00007	MIMAT0000805	rno-miR-30e-5p	0.307	0.01487
MIMAT0000781	rno-miR-9a-5p	1.544	0.00029	MIMAT0000857	rno-miR-181c-5p	0.365	0.01700
MIMAT0000560	rno-miR-326-3p	0.541	0.00062	MIMAT0000863	rno-miR-186-5p	0.301	0.01915
MIMAT0004708	rno-miR-9a-3p	0.996	0.00077	MIMAT0000800	rno-miR-28-5p	0.200	0.01953
MIMAT0000552	rno-miR-301a-3p	0.669	0.00201	MIMAT0000547	rno-miR-322-3p	0.150	0.01955
MIMAT0000821	rno-miR-99b-5p	0.185	0.00329	MIMAT0003198	rno-miR-376a-3p	0.786	0.02026
MIMAT0024854	rno-miR-6215	0.350	0.00372	MIMAT0003114	rno-miR-383-5p	0.316	0.02100
MIMAT0000796	rno-miR-26a-5p	0.380	0.00383	MIMAT0000579	rno-miR-148b-3p	0.361	0.02128
MIMAT0000575	rno-miR-335	0.368	0.00411	MIMAT0001619	rno-miR-322-5p	0.342	0.02178
MIMAT0000826	rno-miR-107-3p	0.354	0.00429	MIMAT0003199	rno-miR-381-3p	0.249	0.02233
MIMAT0000791	rno-miR-22-3p	0.460	0.00495	MIMAT0005321	rno-miR-500-3p	0.241	0.02335
MIMAT0000855	rno-miR-153-3p	0.574	0.00539	MIMAT0005339	rno-miR-873-5p	0.152	0.02414
MIMAT0000842	rno-miR-136-5p	0.738	0.00586	MIMAT0000794	rno-miR-24-3p	0.247	0.02519
MIMAT0000812	rno-miR-33-5p	0.190	0.00610	MIMAT0005325	rno-miR-598-3p	0.321	0.02694
MIMAT0003383	rno-miR-497-5p	0.500	0.00635	MIMAT0004720	rno-miR-30e-3p	0.296	0.02763
MIMAT0000801	rno-miR-29b-3p	0.459	0.00758	MIMAT0005323	rno-miR-532-3p	0.164	0.02880
MIMAT0000806	rno-miR-30b-5p	0.247	0.00759	MIMAT0005282	rno-miR-872-5p	0.193	0.02885
MIMAT0005596	rno-miR-551b-3p	0.372	0.00788	MIMAT0000799	rno-miR-27a-3p	0.303	0.03087
MIMAT0000553	rno-miR-324-5p	0.461	0.00814	MIMAT0005322	rno-miR-532-5p	0.176	0.03287
MIMAT0000615	rno-miR-101b-3p	0.410	0.01005	MIMAT0000570	rno-miR-331-3p	0.188	0.03329
MIMAT0000877	rno-miR-204-5p	0.477	0.01066	MIMAT0000829	rno-miR-125a-5p	0.168	0.03635
MIMAT0000820	rno-miR-99a-5p	0.293	0.01115	MIMAT0000574	rno-miR-140-3p	0.204	0.03784
MIMAT0012833	rno-miR-582-5p	0.220	0.01237	MIMAT0003116	rno-miR-501-5p	0.884	0.04308
MIMAT0000573	rno-miR-140-5p	0.407	0.01283	MIMAT0000587	rno-miR-341	0.484	0.04634
MIMAT0003178	rno-miR-542-5p	0.191	0.01377	MIMAT0003179	rno-miR-542-3p	0.213	0.04869
MIMAT0000823	rno-miR-101a-3p	0.409	0.01401	MIMAT0017307	rno-miR-434-5p	0.191	0.04946
MIMAT0000870	rno-miR-195-5p	0.389	0.01430	MIMAT0001547	rno-miR-450a-5p	0.149	0.04992
*Downregulated*							
MIMAT0024856	rno-miR-6216	−1.375	0.00003	MIMAT0000893	rno-miR-290	−0.492	0.01830
MIMAT0012827	rno-miR-1224	−0.718	0.00026	MIMAT0000885	rno-miR-214-3p	−0.489	0.02027
MIMAT0024853	rno-miR-3473	−0.736	0.00568	MIMAT0017807	rno-miR-3549	−0.189	0.02202
MIMAT0005278	rno-miR-466b-5p	−0.863	0.00802	MIMAT0025065	rno-miR-6326	−0.178	0.02346
MIMAT0005301	rno-miR-188-5p	−0.249	0.00864	MIMAT0017840	rno-miR-3065-3p	−0.364	0.02438
MIMAT0035732	rno-miR-1896	−0.818	0.01267	MIMAT0017133	rno-miR-150-3p	−1.148	0.02654
MIMAT0025048	rno-miR-3099	−0.861	0.01347	MIMAT0005337	rno-miR-760-3p	−0.308	0.04226
MIMAT0003382	rno-miR-664-3p	−0.593	0.01739	MIMAT0017305	rno-miR-423-5p	−0.545	0.04541

### miRNA-mRNA target predictions

**Figure 2:**
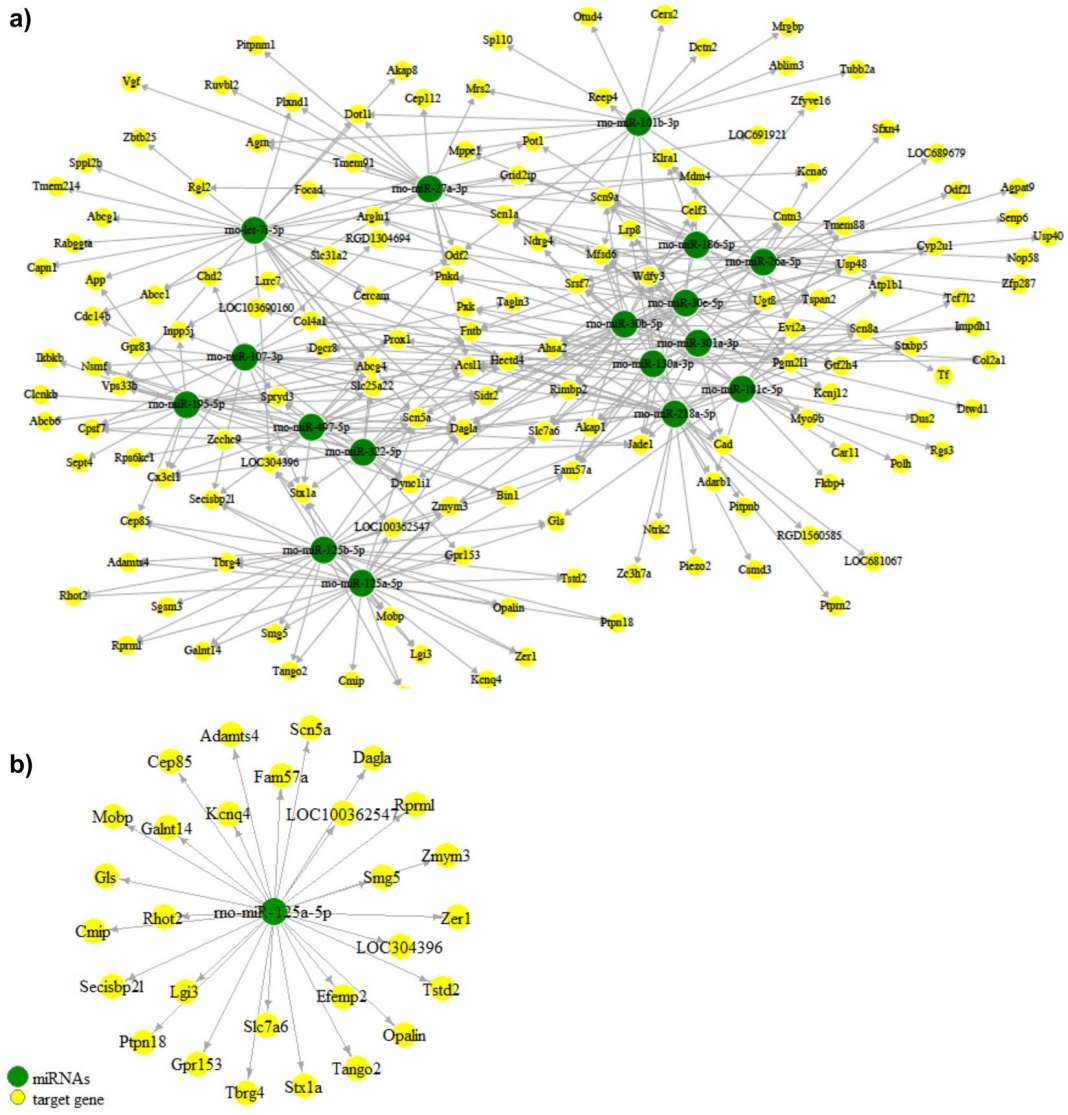
Predicted miRNA-mRNA target network. (**a**) Network of predicted and experimentally validated miRNA-mRNA target interactions using inversely correlated DEG targets and their DEmiR with highest degree of connectivity. (**b**) rno-miR-125a-5p is predicted to target the most number of genes within our DEG results using negative correlation.

MultimiR^33,34^ was used to find validated and predicted miRNA-gene targets using inversely regulated DEmiRs and DEGs. Considering the top 20% of predicted scores and conserved targets sites, 618 unique miRNA-gene target pairs were found with 290 nodes (58 miRNAs and 232 genes) based on conserved prediction sites. The majority of the miRNA-gene pairs were from upregulated DEmiRs and downregulated DEGs. Pair-wise Pearson correlation analysis was performed on expression values of DEGs and DEmiR. Multiple testing was corrected using BH method. Figure 2a shows the predicted network for miRNA-mRNA in VTA DA neuron after perinatal nicotine exposure. Notably, rno-miR-125a-5p was predicted to target the most number of unique genes (Fig. 2b).

### Enriched pathway analysis and integrated miRNA-mRNA network

According to DAVID, pathway analysis of the downregulated DA DEGs revealed significant enrichment of many Kyoto Encyclopedia of Genes and Genomes (KEGG) pathways associated with addiction, including neuroactive ligand-receptor interaction (*p*  ≪ 0.001), calcium signaling (*p*  ≪ 0.001), cAMP signaling (*p* < 0.01), and long-term potentiation (*p* < 0.05). Additionally, multiple signaling pathways and synapse pathways were enriched as well as drug addiction pathways (see Table 3 for more details). Notably, the DAergic synapse pathway was significantly enriched (*p* ≪ 0.001). Significant enrichment was determined using a modified hypergeometric test. Upregulated DA DEGs also showed non-significant enrichment of glutamatergic and serotonergic synapse pathways and neuroactive ligand-receptor interaction pathway.

**Table 3:** KEGG pathways enriched by down or upregulated DEGs and the corresponding genes identified in pathway analysis.

KEGG Term	*p* value	Genes
*Downregulated*		
Neuroactive ligand-receptor interaction	8.56E-06	Hcrtr1, Grik2, S1pr2, Gabrg2, Htr4, Tacr3, Sstr3, Adra1b, Gabrd, Grik3, Grin2d, Galr2, Gria3, Chrnb4, Chrm3, Grm4
Insulin secretion	1.59E-04	Camk2g, Stx1a, Atp1b1, Kcnmb4, Kcnn1, Kcnn2, Chrm3
Dopaminergic synapse	3.55E-04	Mapk8, Caly, Camk2b, Scn1a, Camk2g, Ppp1ca, Gsk3b, Itpr1, Gria3
Calcium signaling pathway	9.03E-04	Camk2b, Camk2g, Grin2d, Itpr1, Htr4, Cacna1g, Cacna1h, Tacr3, Adra1b, Chrm3
Aldosterone synthesis and secretion	9.54E-04	Camk1g, Camk2b, Camk2g, Itpr1, Camk1, Cacna1g, Cacna1h
African trypanosomiasis	0.001683	LOC689064, Hbb, Hbb-b1, LOC100134871, Hba2
Amphetamine addiction	0.001721	Camk2b, Camk2g, Ppp1ca, Grin2d, Stx1a, Gria3
Circadian entrainment	0.002012	Camk2b, Camk2g, Grin2d, Itpr1, Gria3, Cacna1g, Cacna1h
Cholinergic synapse	0.004135	Camk2b, Camk2g, Kcnj12, Kcnq4, Itpr1, Chrnb4, Chrm3
Glutamatergic synapse	0.004705	Grik2, Grin2d, Itpr1, Gria3, Gls, Grm4, Grik3
cAMP signaling pathway	0.004999	Mapk8, Camk2b, Camk2g, Ppp1ca, Grin2d, Gria3, Htr4, Atp1b1, Pak1
Oxytocin signaling pathway	0.006062	Camk1g, Camk2b, Camk2g, Ppp1ca, Kcnj12, Map2k5, Itpr1, Camk1
Malaria	0.008344	LOC689064, Hbb, Hbb-b1, LOC100134871, Hba2
Long-term potentiation	0.011670	Camk2b, Camk2g, Ppp1ca, Grin2d, Itpr1
Nicotine addiction	0.016858	Grin2d, Gabrg2, Gria3, Gabrd
Gastric acid secretion	0.017274	Camk2b, Camk2g, Itpr1, Atp1b1, Chrm3
ErbB signaling pathway	0.035280	Mapk8, Camk2b, Camk2g, Gsk3b, Pak1
Alzheimer’s disease	0.037631	Atp5b, Gsk3b, Grin2d, Atp5c1, Itpr1, App, Aph1b
*Upregulated*		
Inflammatory mediator regulation of TRP channels	0.013087	Il1r1, Adcy2, Cyp2c7, Htr2a, Trpv3
HTLV-I infection	0.027675	Il1r1, Pdgfa, Adcy2, Pold4, RT1-N2, RT1-A3, Kat5
Pancreatic secretion	0.040812	Slc4a2, Pla2g2c, Adcy2, Clca4l
Choline metabolism in cancer	0.046302	Chpt1, Pdgfa, Slc22a4, Pcyt1b
Glutamatergic synapse	0.063499	Adcy2, Grin2d, Gng4, Slc1a6
Serotonergic synapse	0.075909	Htr6, Cyp2c7, Gng4, Htr2a
Neuroactive ligand-receptor interaction	0.082295	Glra1, Htr6, Grin2d, Htr2a, Chrna1, Hrh4

**Figure 3:**
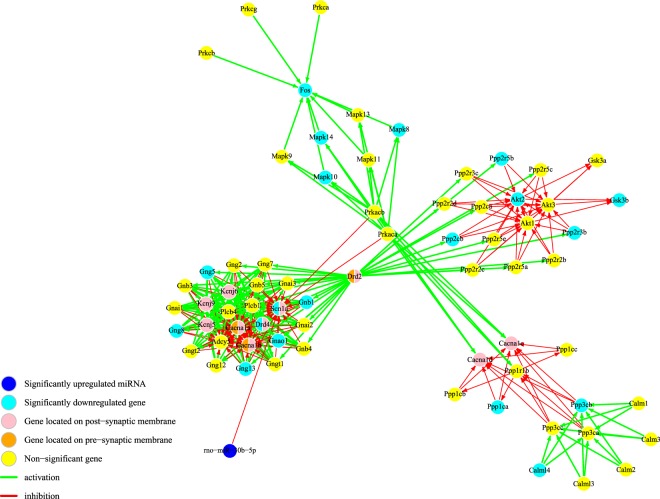
Enriched KEGG dopaminergic synapse pathway following perinatal nicotine exposure in DA neurons. KEGG’s DAergic synapse pathway describes the release of the neurotransmitter DA from the presynaptic (orange) to the postsynaptic (pink) neuron. miR-30b-5p (dark blue) was predicted to target Scn1a, a protein-coding gene involved in voltage-gated sodium channels, and had significant predicted interaction with the DAergic synapse pathway (p < 0.01). Light blue nodes are significantly downregulated DEGs in the results and yellow nodes are genes that were not detected or not significantly in the results. Genes with multiple implications have been multi-colored.

**Figure 4:**
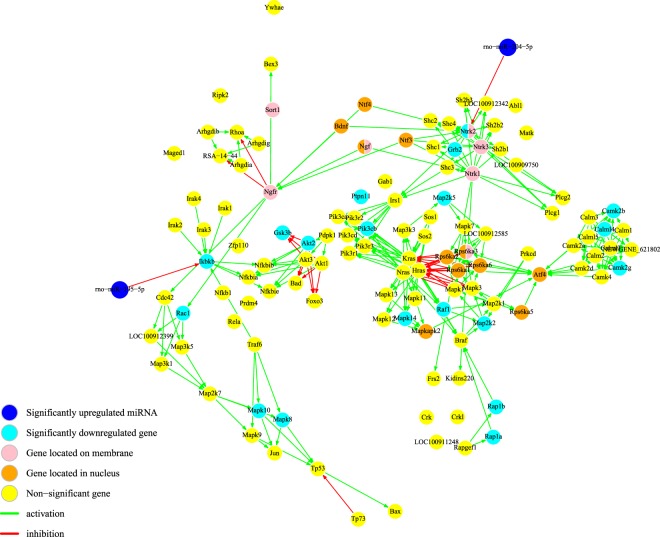
Enriched KEGG neurotrophin signaling network following perinatal nicotine exposure in DA neurons. The neurotrophin signaling pathway describes the activation of multiple intracellular signal transduction pathways through the binding of Ntrk2 (TrkB) to Bdnf on the cell membrane. Our results predicted that Ntrk2 was targeted by miR-204-5p (dark blue). Additionally, rno-miR-195-5p (dark blue) was predicted to target Ikbkb. The predicted interaction of both miRNAs on the neurotrophin signaling pathway was significant (p < 0.01).Light blue nodes are significantly downregulated DEGs in the results and yellow nodes are genes that were not detected or not significantly in the results. In addition to significance, location of the mRNA has been indicated by color (pink for membrane, orange for nucleus, otherwise cytoplasm).

A gene network was created using KEGGgraph^35^ incorporating enriched pathways found using DAVID^36,37^. Additional relevant KEGG pathways were included from the literature^18,21^. A subset graph was created removing genes that were not significant in the microarray results in order to visualize gene-gene interactions within our results. Results from miRNA-gene target predictions were included in order to show the relationship between genes and pathways and their corresponding miRNA predictions. Figures 3 and 4 show the mRNA network created from the dopaminergic synapse pathway and neurotrophin signaling pathway, respectively, with miRNA targeting interaction. According to the hypergeometric test, we found two pathways with significant miRNA-gene target prediction with significant miRNA-pathway interaction results: DAergic synapse pathway and rno-miR-30b-5p was (*p* < 0.01) and the neurotrophin signaling pathway and rno-miR-195-5p and rno-miR-204-5p (*p* < 0.01).

### Validation of miRNA and mRNA microarray results using RT-qPCR

**Figure 5:**
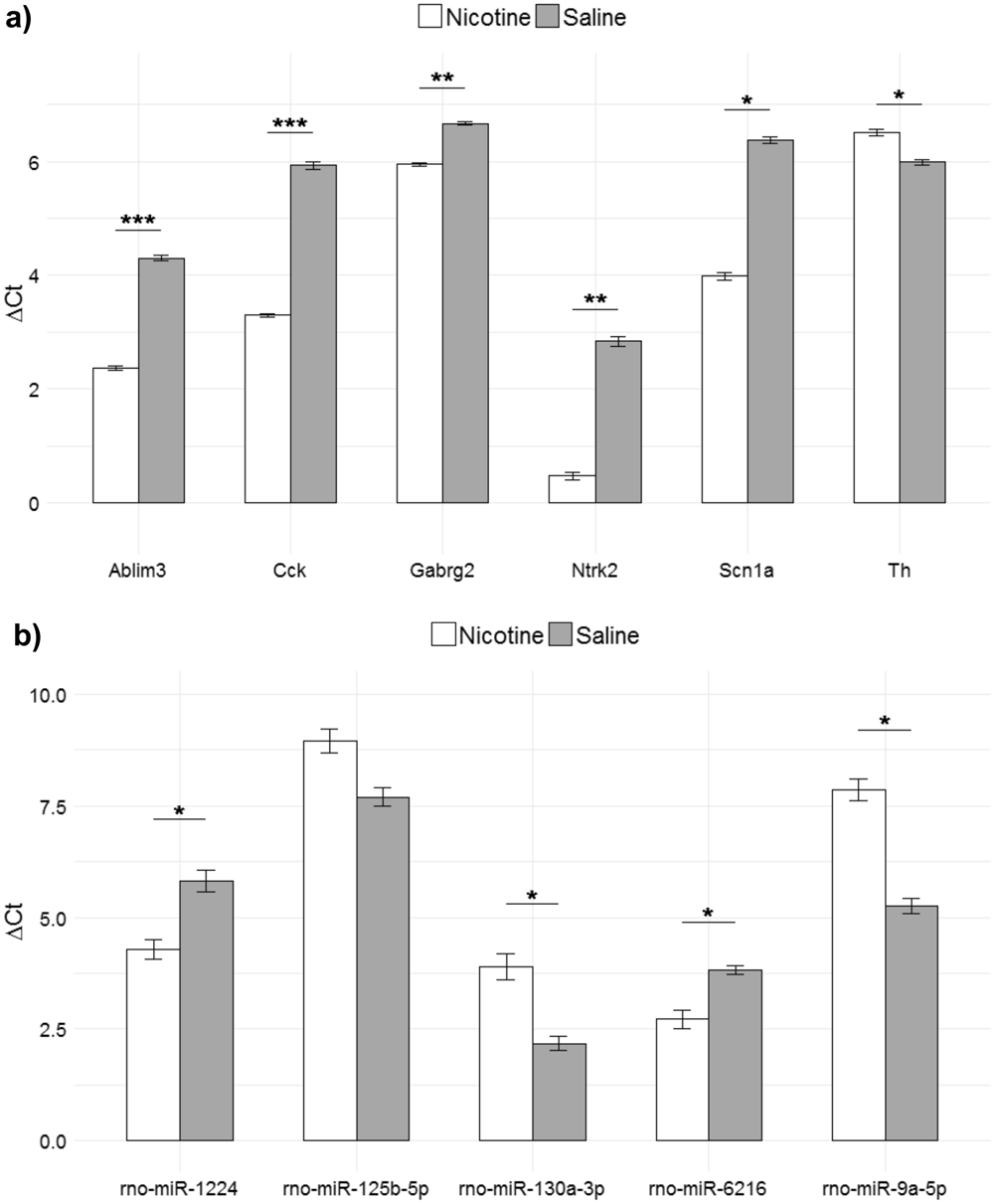
RT-qPCR validation results of microarray data comparing gene (top) and miRNA (bottom) expression in DA neurons in the VTA comparing perinatal exposure to nicotine and saline control. Six DEGs (top) were tested and five DEmiRs to assess validity of microarray experiments using the same total RNA sample used in microarray experiments. The results from RT-qPCR validation experiments are shown as ΔCt values relative to control: GAPDH for mRNA (top) and U6 snRNA for miRNA (bottom) experiments. Significance was evaluated using Student’s t-test (n = 3) and corrected for multiple comparisons using Benjamini-Hochberg procedure with false discovery rate of 0.05 (*p < 0.05, **p < 0.01).

The microarray results were validated for both miRNA and mRNA using the same approach as detailed in Bosch *et al*.^29^. Candidate genes were selected based on previously reported gene expression profiling of DA neurons in the VTA and biological interest from pathway enrichment and miRNA-gene target prediction. Significant miRNAs and mRNAs were selected for further analysis using RT-qPCR in order to validate the results of the microarrays. For mRNA microarray validation, two previously reported DA neuron markers were chosen, Cck and Gabrg2, as well as the significant DEGs found in the integrated miRNA-gene network, Scn1a, Ntrk2, and Ablim3. The direction of regulation resulting from RT-qPCR was consistent with the microarray results from our experiments, indicating that the data produced by the microarray experiments were valid. Regulation was determined using Student’s t-test comparing nicotine and saline treatment groups and corrected for multiple testing using the BH method with false discovery rate of 0.05 (Fig. 5).

## Discussion

The current study is the first to conduct a large-scale profile of both miRNA and mRNA expression in DA neurons located in the VTA of rats following perinatal nicotine exposure. In our current work, the offspring was exposed to nicotine over a period equivalent to the three trimesters of human pregnancy^4,13^. Our study sought to identify significant genes and miRNAs in DA neurons modulated by perinatal nicotine exposure. After differential expression analysis, our results showed 1,774 DEGs were downregulated and 862 DEGs were upregulated. Additionally, 16 DEmiRs were downregulated and 58 DEmiRs were upregulated. The gene expression profile of DA neurons was analyzed using DAVID for functional enrichment analysis. Enriched KEGG pathways were analyzed in order to summarize putative processes modulated by perinatal nicotine exposure compared to saline. In addition, target predictions for DEmiRs were compared with DEG results in order to identify miRNA-gene pairs that may be changed after perinatal nicotine exposure. Further analysis of these results revealed potential points of pathway regulation by DEmiRs that may be involved in altered mechanisms following perinatal nicotine exposure.

Many gestational or perinatal nicotine exposure studies have focused on nicotinic acetylcholine receptors (nAChRs), which were reduced following gestational nicotine exposure in adolescent rats. One study exposed rats to nicotine during fetal and neonatal brain development (similar to the time of exposure used in our study)^13^. They found that during P7-14, the number of high-affinity nAChRs reach their highest levels in most brain regions^13^, which is the time period of our experiment. They also found decreased expression of multiple nAChR subunits including β4 mRNA subunit (Chrnb4), which we found significantly reduced as well. Additionally, Chrnb4 is involved in the neuroactive ligand-receptor interaction and cholinergic synapse pathways, which were significantly enriched by downregulated DEGs. The β4 subunit is only expressed in about 10% of DA neurons with a much higher expression in GABA neurons, but the time point of our study and equivalent amount of DA neuron total RNA may account for the expression pattern of nAChRs found in this experiment. Further studies focusing specifically on the nAChR subunits are necessary in order to determine if nAChR mRNA expression is decreased due to nicotine exposure or if reduced expression is caused by decreased DA neuron population.

Notably, the nicotine addiction KEGG pathway was enriched from our downregulated DEGs and four genes were found to be significant among our results. The enrichment of the nicotine addiction pathway indicates that there may be common genetic alterations in the brain that occur between gestational nicotine-treated offspring and adults with nicotine dependence. The first two genes, Gabrd and Gabrg2, are subunits of the γ-Aminobutyric acid (GABA) A receptor which were both significantly downregulated in our results. Our microarray tested the α-, β-, and θ-subunits of the GABA-A receptor, but these were not significantly differentially expressed in our results. A recent study by Stojakovic *et al*. investigated the selective deletion of Gabrg2 on DA neurons. Mice with the deletion showed a high risk of developing alcohol addiction based on behavior and an increased conditioned place preference to alcohol^38^. These results suggest that despite the expression of other subunits of the GABA-A receptor, Gabrg2 expression is important in DA neurons and DA neurotransmission in response to drugs. Another study investigating DA neurons in the VTA following cocaine exposure found GABA-A receptor subunits, including Gabrg2, to be significantly downregulated^17^. While this study did not use a model of gestational drug exposure, it focused on alterations within VTA DA neurons following chronic drug exposure.

The last two genes were downregulated in our results and encode subunits of glutamate receptors. Grin2d is the 2D subunit of N-methyl-D-aspartate (NMDA) receptors, which are a class of glutamate ionotropic receptors. Gria3 is subunit 3 of the alpha-amino-3-hydroxy-5-methyl-4-isoxazole propionate (AMPA) type glutamate ionotropic receptors. A study by Roguski *et al*. investigated intra-VTA NMDA receptors and concluded that a subpopulation of NMDA receptors were responsible for increased vulnerability to DA release in NAc. In addition, previous studies indicated that the expression of NMDA subunits was unchanged due to chronic ethanol exposure followed by withdrawal, but the function of the receptor was changed^11^. Further investigation needs to be conducted on all receptor subunits in order to determine if GABA and glutamate receptors on DA neurons are significantly modulated by perinatal nicotine exposure.

To date, a vast body of research has been conducted investigating the implication of miRNAs in addiction using different drugs of abuse. However, our present study investigates, for the first time, the large-scale effects of miRNAs in VTA DA neurons after perinatal nicotine exposure. We recently reported the upregulation of miR-140-5p after perinatal nicotine exposure. From our miRNA expression results, miR-140-3p and miR-140-5p were both significantly differentially expressed and have both been implicated in nicotine addiction^26^. Huang *et al*. found that miR-140-3p expression was increased by nicotine, which then repressed the expression of dynamin 1 (Dnm1) by direct base-pairing to the 3′-untranslated region of the gene^39^. In accordance with their results, miR-140-3p was significantly upregulated and Dnm1 was downregulated in our results, although not significantly. In our previous study, we investigated the modulation of addiction-related miRNAs by nicotine and found upregulation of miR-140-5p, which we confirmed with our current results^26^. Further studies should be conducted on miR-140-3p and miR-140-5p and their potential gene targets in order to elucidate their relationship to nicotine exposure.

The miRNA-gene network indicated that miR-125a-5p was the most highly predicted miRNA based on DEG targets (Fig. 2b). A study by Bosch and colleagues identified miR-125a-5p as an addiction-related miRNA in a study investigating the changes in RNA expression in the VTA due to the self-administration of methamphetamine in rats^29^. Their study found an overall upregulation of miR-125a-5p in the VTA^29^, which confirms the upregulation of miR-125a-5p in VTA DA neurons in our results. The significance of miR-125a-5p in the VTA in both of these studies implicates its role in regulating pathways related to addictive drugs, including methamphetamine and nicotine. Further study into the genes targeted by miR-125a-5p could provide a clearer picture of the precise nature of this miRNA in pathways related to substance abuse.

Our integrated network of DEGs and DEmiRs was created from the enriched KEGG pathways with the addition of significantly upregulated predicted miRNA in order to visualize putative points of pathway regulation by miRNAs. These pathways were enriched by the downregulated DEGs indicating important putative pathways for miRNA regulation as tested by hypergeometric testing with correction for multiple testing. Significantly upregulated predicted miRNAs were added to this network in order to visualize points of pathway regulation by miRNA. In the DAergic synapse pathway, miR-30b-5p was predicted to target Scn1a, a protein-coding gene involved in voltage-gated sodium channels (Fig. 3). As a whole interaction network, the probability of the involvement of miR-30b-5p in the DAergic synapse pathway was significant (*p* < 0.01). The enrichment of this pathway supports the hypothesis that the mesocorticolimbic DA pathway is involved in the reinforcing effects of substance abuse. The DAergic synapse pathway describes the release of DA neurotransmitter from DA neurons to a postsynaptic neuron. According to the major hypothesis of drug reinforcement, the reinforcing effect of addiction is believed to be conveyed through the activation of the mesocorticolimbic DA system. Stimulation of VTA DA neurons via nicotine administration results in the release of DA in the NAc, describing the role of the DA synapse pathway in the reinforcing effect^7^. The enrichment of this pathway in our results demonstrates that perinatal nicotine exposure leads to genetic alterations in VTA DA neurons that are in accordance with addiction mechanisms. In addition, we have highlighted a potential gene-miRNA target interaction, which may contribute to the observed alterations via post-transcriptional gene regulation.

The neurotrophin signaling was enriched in our results at a significance *p* < 0.1. Our analysis revealed that Ntrk2 (also known as TrkB) was predicted to be targeted by miR-204-5p and is involved in the neurotrophin signaling pathway and miR-195-5p was predicted to target Ikbkb (Fig. 4). As a whole interaction network, the probability of the involvement of both miRNAs in the neurotrophin signaling pathway was significant (*p* < 0.01). Many studies have been conducted linking nicotine and the neurotrophin signaling pathway, but do not include the VTA DA neurons^28^. TrkB, a transmembrane receptor with a high affinity for tyrosine kinaseB, is activated by Bdnf (brain-derived neurotrophic factor), which has been reported to potentiate the effects of addictive substances through the mesocorticolimbic DA pathway^40,41^. Additionally, TrkB has been identified as a susceptibility gene for psychiatric disorders, such as schizophrenia and other mood and anxiety disorders. TrkB plays a role in synaptic plasticity and neurotransmitter release and may provide additional support for the alterations induced by nicotine in DA neurons in the VTA^42^.

In summary, we focused on the response of VTA DA neurons to perinatal nicotine exposure and investigated transcriptional and post-transcriptional regulation disruptions. We also concentrated on the identification of biological pathways modulated by nicotine within the DA neurons of the VTA. Our study suggested that the dopaminergic synapse pathway was significantly altered by perinatal nicotine exposure as well as significant interactions with miRNAs. Although our results indicate that perinatal nicotine exposure alters the expression of miRNAs and genes, demonstrating the involvement of several biological pathways in the mechanism of addiction in DA neurons of the VTA, we did not investigate the effect of gender on DA neurons in the VTA following perinatal nicotine exposure in the current study. Such an investigation merits further exploration as there is evidence indicating nicotine exposure differentially affects gene expression changes in many different regions of the brain depending on gender^43^. Additionally, further investigation needs to be performed to develop an interactive model to evaluate the effect of miRNAs on biological pathways.

## Materials and Methods

### Animal treatment

All experiments were performed in accordance with the protocols and surgical procedures that were approved by the Institutional Animal Care and Use Committee (IACUC) and the University of Houston Animal Care Operations (ACO). Pregnant female Sprague–Dawley (SD) rats (Charles River, Wilmington, MA, USA) were maintained on a 12-h light/12-h dark schedule at a temperature of 22 ± 2 °C and 65% humidity. Access to standard food and water was ad libitum. Rats were acclimated to the animal facility for 72 hours before they received treatment via an osmotic minipump (Alzet, Cupertino, CA) that was implanted subcutaneously containing either nicotine hydrogen tartrate (Sigma-Aldrich, St. Louis, MO, USA) released at a rate of 6 mg/kg/day (moderate to heavy smokers^5,12^), or an equal volume of saline vehicle for the control. Nicotine continues to be released from the osmotic minipump for 4 weeks from gestational day 6 to postnatal day 14.

Seven-to-fourteen day-old pups (male and female) were anesthetized with isoflurane before decapitation. On a VT1200 semiautomatic vibrating blade microtome (Leica, Nussloch, Eisfeld, Germany), horizontal slices containing VTA were cut at a thickness of 1000 μm. Brain punches containing the VTA were collected bilaterally using a 1 mm biopsy punch (Integra Miltex, VWR, Radnor, PA, USA) and placed in 1 mL of Hibernate A (Gibco, Thermo Fisher Scientific, USA) on ice to maintain cell viability. Brain punches were pooled so that one litter resulted in one litter. A total of four samples for both saline and nicotine treated groups were processed for RNA extraction and microarray processing.

### Cell Dissociation, FACS, and RNA extraction

Tissue was dissociated into a single cell solution and sorted by FACS as reported by Guez-Barber *et al*.^32^. Briefly, tissue punches were placed in 1 mL of Accutase (Gibco, Thermo Fisher Scientific, USA) and shaken for 30 minutes at 4 °C in order to dissociate the tissue punches. Accutase is a mixture of proteolytic and collagenolytic enzymes, which showed to produce the most single cells with the least cell damage^32^. Cells were pelleted at 425 × *g* and resuspended in Hibernate A. To further dissociate cells, cells were gently pipetted with increasingly smaller pipette tips. The supernatant containing single cells was collected until all cells were collected. Then, to remove debris and cell clusters, the cell suspension was serially filtered through pre-wetted 100 µm and 40 µm cell strainers. The strained cell suspension was added to a three-density step gradient made using Percoll (GE Healthcare, VWR, USA) and centrifuged at 430 × *g* for 3 minutes in order to further remove debris. The cloudy top layer containing debris was removed and remaining solution was centrifuged at 550 × *g* for 5 minutes to pellet the cells. The cells were fixed for immunolabeling by resuspending them in equal parts Hibernate A and 100% cold ethanol, gently vortexed, and kept on ice for 15 minutes. Cells were co-labeled with conjugated primary antibodies neuronal marker, NeuN/Alexa Fluor 488 (NeuN/AF488, ab190195, Abcam, Cambridge, MA, USA), and tyrosine hydroxylase/phycoerythrin (TH/PE, ab209921, Abcam, Cambridge, MA, USA). Using conjugated antibodies simplifies the staining procedure by eliminating the need for a secondary antibody, which can bind unspecifically and reduces the time it takes to stain cells.

An Influx (BD Biosciences, San Jose, CA, USA) instrument was used to sort the cells at the Flow Cytometry and Cellular Imaging Core Facility (MD Anderson – South Campus, Houston, TX, USA). Samples were sorted based on double-positive NeuN^+^/TH^+^ staining. Once sorted, cells were centrifuged into a pellet at 2650 × *g* for 8 minutes at 18 °C. Total RNA was isolated using miRNeasy Micro Kit (Qiagen, Hilden, Germany) following manufacturer’s instructions, including DNAse treatment and used in subsequent microarray and RT-qPCR validation experiments. RNA quality and quantity was established according to the optical density (OD) of each sample at 260 nm and 280 nm determined using a NanoDrop 2000 spectrophotometer (Thermo Fisher Scientific, Waltham, MA, USA). Only total RNA samples with A260/280 ratio of 1.9 or greater were used in subsequent experiments.

### mRNA and miRNA expression microarrays

All gene and miRNA expression reagents and kits were purchased from Agilent (Santa Clara, CA, USA) unless otherwise stated. The gene expression was profiled using a SurePrint G3 Rat Gene Expression v2 8 × 60 K microarray (ID: 074036) with 30,584 unique genes. With 25 ng of total RNA, samples were prepared using the One-Color Low Input Quick Amp Labeling kit with RNA Spike-Ins according to manufacturer’s instructions. First, total RNA was amplified and labeled with Cyanine-3 (Cy3). The amplified cRNA was purified and quantified using a NanoDrop 2000 spectrophotometer. Next, 600 ng of Cy 3-labeled cRNA with required specific activity (≥6 pmol Cy3/µg cRNA) were fragmented and prepared for hybridization using the Gene Expression Hybridization kit. Then, slides were hybridized for 17 hours at 65 °C and washed in Gene Expression Wash Buffers according to manufacturer’s protocol.

For miRNA expression, an 8 × 15 K Rat miRNA Microarray, Release 21.0 (ID: 070154) was used containing 758 mature miRNAs. For labeling and hybridization, miRNA Complete Labeling and Hyb kit with RNA Spike-Ins was used according to manufacturer’s instructions. RNA Spike-In was included to test quality control of the microarray amplification, labeling, and hybridization. Briefly, 100 ng of total RNA containing miRNAs was dephosphorylated and labeled with Cy3-pCp. Samples were purified on a Micro Bio-Spin P-6 gel column (Bio-Rad), dried, and hybridized at 55 °C for 20 hours. Microarray slides were washed using Gene Expression Wash Buffers according to manufacturer’s protocol.

The gene and miRNA expression slides were scanned using G4900DA SureScan Microarray Scanner using G3_GX_1color and AgilentG3_miRNA scan protocols, respectively. Microarray data was extracted from the TIFF result images using Feature Extraction (FE) Software v12.0.1.1 and the corresponding protocol, GE1_1200_Jun14 FE protocol for gene expression and miRNA_1200_Jun14 protocol for miRNA expression.

### Data Analysis

All pre-processing, normalization, and statistical analyses were performed using several Bioconductor packages in R version 3.4.2^44^. The quality of microarray data was assessed using arrayQualityMetrics^45^ package. Raw mRNA expression data was imported using limma^46^ package. Before normalization, probes expressing Agilent flags for feature and background outliers were removed from the analysis. Raw median intensity values were background corrected using “normexp” method and quantile normalized. Control probes were filtered out as well as low expressed probes, which were defined by expression intensity less than 10% brighter than 95% intensity of negative controls. Replicated probes were averaged and a total of 27,791 genes were selected for analysis. Quality assessment was performed to check for outliers among microarrays. A linear fit model was used to calculate fold changes and standard errors for each gene of interest. To increase the power of the data, standard errors were moderated using the eBayes function, which applies a simple empirical Bayes model and computes a log-odds of differential expression for each contrast. P-values were corrected for multiple testing using BH method. DEGs were identified using a threshold of *q* < 0.01 and absolute log fold change >1.

For miRNA microarray data, raw intensity data was imported and processed using AgiMicroRna^47^ package. Quality of the microarray was assessed using arrayQualityMetrics package as well as quality control functions within the AgiMicroRna package. Raw miRNA data was preprocessed using the robust multi-array average (RMA) algorithm without background correction. Data was filtered according to the following criteria: (1) positive for flag IsGeneDetected, (2) expressed in at least 50% of the experimental samples, and (3) signal intensity greater than the mean value of the negative control + 1.5 standard deviations. After filtering, 329 miRNAs remained for analysis. Linear model was fitted to the miRNA expression data and moderated statistics calculated using eBayes, similar to mRNA data analysis. Differential expression was identified using *q*-value threshold <0.05 (calculated using BH method)^28,29^.

### Integrated analysis of miRNA-mRNA

Functional enrichment analysis was performed on differentially expressed genes using DAVID v6.8^36,37^. Enriched KEGG pathways were identified and analyzed. Further analysis of KEGG pathways was done using DEgraph^48^ and KEGGgraph^35^. MultiMiR^33,34^ was used to identify predicted and validated miRNA-gene target pairs based on inversely correlated regulation of DEmiRs and DEGs. multiMiR is a comprehensive compilation of predicted and validated miRNA-gene target interactions from 14 external databases. Hypergeometic testing was performed on target DEGs negatively correlated with DEmiRs using pairwise Pearson correlation analysis and then BH corrected for multiple comparisons^49^. Remaining significant miRNA-gene target pairs were integrated into gene network models based on enriched KEGG pathways. Following the procedure used in the miRPathDB^50^, we performed a hypergeometric test with multiple testing correction using BH method and an FDR < 0.05 for each miRNA-gene predicted interaction. miRPathDB is a tool to measure the association between miRNAs and putative target pathways.

### Quantitative RT-PCR validation of microarray data

All reagents and kits for quantitative reverse transcription polymerase chain reaction (RT-qPCR) were purchased from Applied Biosystems (Thermo Fisher Scientific, Carlsbad, CA, USA) unless otherwise stated. Seven genes and five miRNAs were chosen for validating microarray data using RT-qPCR. Total RNA was isolated from FACS samples for each experimental group as described above. For gene expression validation, cDNA was prepared using High Capacity cDNA Reverse Transcription Kit according to manufacturer’s instructions. For miRNA expression validation, cDNA was prepared and preamplified using TaqMan Advanced miRNA cDNA Synthesis Kit according to manufacturer’s instructions. For gene validation and miRNA validation, quantitative PCR (qPCR) was carried out using TaqMan Fast Advanced Master Mix and corresponding TaqMan Assay (TaqMan Gene Expression Assay or TaqMan Advanced miRNA Assay) on a StepOnePlus Real-Time PCR System according to manufacturer’s instructions using the following parameters: 2 min at 50 °C, 2 min at 95 °C, 40 cycles of 1 sec at 95 °C and 20 sec at 60 °C. Each reaction was prepared in triplicate for both validation sets. Comparative Ct method was used to find the relative quantity of the target genes or miRNAs. Student’s t-test was performed comparing nicotine and saline treatment groups (n = 3) and corrected for multiple testing using the BH method with false discovery rate of 0.05.
